# Measuring Women's Empowerment in Sub-Saharan Africa: Exploratory and Confirmatory Factor Analyses of the Demographic and Health Surveys

**DOI:** 10.3389/fpsyg.2018.00994

**Published:** 2018-06-19

**Authors:** Ibitola O. Asaolu, Halimatou Alaofè, Jayleen K. L. Gunn, Akosua K. Adu, Amanda J. Monroy, John E. Ehiri, Mary H. Hayden, Kacey C. Ernst

**Affiliations:** ^1^Department of Health Promotion Sciences, Mel and Enid Zuckerman College of Public Health, University of Arizona, Tucson, AZ, United States; ^2^Department of Epidemiology and Biostatistics, Mel and Enid Zuckerman College of Public Health, University of Arizona, Tucson, AZ, United States; ^3^Department of Epidemiology, College of Public Health, University of Kentucky, Lexington, KY, United States; ^4^School of Geography and Development, University of Arizona, Tucson, AZ, United States; ^5^Climate Science and Applications Program, National Center for Atmospheric Research, Boulder, CO, United States

**Keywords:** women's empowerment, gender equality, exploratory factor analysis, confirmatory factor analysis, Sub-Saharan Africa

## Abstract

**Background:** Women's status and empowerment influence health, nutrition, and socioeconomic status of women and their children. Despite its benefits, however, research on women's empowerment in Sub-Saharan Africa (SSA) is limited in scope and geography. Empowerment is variably defined and data for comparison across regions is often limited. The objective of the current study was to identify domains of empowerment from a widely available data source, Demographic and Health Surveys, across multiple regions in SSA.

**Methods:** Demographic and Health Surveys from nineteen countries representing four African regions were used for the analysis. A total of 26 indicators across different dimensions (economic, socio-cultural, education, and health) were used to characterize women's empowerment. Pooled data from all countries were randomly divided into two datasets—one for exploratory factor analysis (EFA) and the other for Confirmatory Factor Analysis (CFA)—to verify the factor structure hypothesized during EFA.

**Results:** Four factors including attitudes toward violence, labor force participation, education, and access to healthcare were found to define women's empowerment in Central, Southern, and West Africa. However, in East Africa, only three factors were relevant: attitudes toward violence, access to healthcare ranking, and labor force participation. There was limited evidence to support household decision-making, life course, or legal status domains as components of women's empowerment.

**Conclusion:** This foremost study advances scholarship on women's empowerment by providing a validated measure of women's empowerment for researchers and other stakeholders in health and development.

## Introduction

Women's empowerment and gender equality concepts are important in fostering health and human development. Empowerment describes the process of change wherein an individual with prior inability to choose has the access and freedom to make choices (Kabeer, 2005). Gender equality is achieved when both men and women enjoy the same socio-economic rights and opportunities and have equal access to education, health care, decent work, and representation in political and economic decision-making processes (World Bank, 2012). Gender equality can be effectively achieved through empowerment, comprising three broad categories, namely (1) agency, which describes the ability to make decisions regardless of existing power relations; (2) resources—including health, education, and physical assets—are the channels through which agency is exercised; and (3) achievements—such as economic opportunities and improved socio-political status—the outcomes of agency (Kabeer, 2005). Over the years, there has been steady improvement in the status of African girls and women, (United Nations, 1979; African Union, 2016) particularly in agency and achievement categories of empowerment (United Nations, 2015). However, identifying ways to track and compare this progress is complicated by the lack of a validated standard method to measure women's empowerment across countries, subnational, and individual levels.

There are currently several indicators that use country-level factors to obtain a measure of empowerment at regional and global levels. Two globally-used indices include the Gender Development Index (GDI)—examining gender differences in human development (health, knowledge, and living standards)—and Gender Inequality Index (GII) assessing gender gaps in reproductive health, empowerment, and labor force participation^1^. Specific indices have also been created for Africa including the African Gender Equality Index (AGEI) and the African Gender and Development Index (UNECA, 2011; African Development Bank, 2015). The AGEI measures gender differences in economic opportunities, human development, and legal rights; the index emphasizes the role of gender equality in advancing agricultural and business productivity (African Development Bank, 2015). Furthermore, the AGDI considers three domains: social power (capabilities), economic power (opportunities), and political power (agency). The AGDI builds upon other indices with the inclusion of the African women's progress scoreboard (AWPS) that details the status of the African woman and girl by considering global treaties that support empowerment (UNECA, 2011). While these indicators have been useful in tracking general progress toward women's empowerment and equality goals, (UNECA, 2011; African Development Bank, 2015)^2^ the data sources limit the ability to examine what individual women are experiencing within the country and fail to demonstrate disparities that may be occurring at the sub-national level.

Individual-level studies have primarily focused on determining the associations between women's empowerment and health care access and outcomes including antenatal care, contraceptive use, child mortality, and nutritional outcomes (Jennings et al., 2014; Heaton, 2015; Phan, 2016; Alaofè et al., 2017; Asaolu et al., 2017). However, studies generally have not incorporated multidimensional indicators of women's empowerment or used an appropriate validation methodology that scientifically corroborates their proposed measures. For example, Jennings et al.'s (2014) study used Demographic and Health Survey (DHS) data from eight African countries to characterize women's empowerment (Jennings et al., 2014). However, the study did not apply a statistical methodology—such as exploratory factor analysis (EFA) or confirmatory factor analysis (CFA)—to identify and validate the latent construct of women's empowerment (Costello and Osborne, 2005; Schreiber et al., 2006). Similarly, the women's empowerment measure developed by Phan (2016) used EFA to describe four domains of women's empowerment across four Southeast Asian countries (Phan, 2016), but the measure was not validated. Furthermore, since the study used data from Southeast Asia, Phan's measure cannot be generalized to African countries given the differences between the continents as exemplified by variations in fertility, educational attainment, and access to improved water and sanitation (World Health Organization, 2015).

Most recently, Ewerling et al. (2017) used DHS data from 34 African countries to create the Survey-Based Women's Empowerment (SWPER) Index which has three domains: attitude to violence, social independence, and decision making (Ewerling et al., 2017). The SWPER indicator is the first developed for broad use across Africa. SWEPR index correlated well with other indicators using national level data, but it has several key limitations. The SWPER's precision may be limited by the application of principal component analysis, and external and convergent validity. The index lacks factor loadings of each domain for individual countries, making it difficult to ascertain the order of domains of women's empowerment across countries. External validation was assessed by correlating SWEPR with the GDI, but these measures of empowerment are distinct. GDI assesses gender equality by calculating the female to male ratio of the human development index (HDI) that comprise specific indicators (i.e. health, knowledge, and living standards), while SWPER used only women's data to describe different indicators of empowerment (i.e. attitudes to violence, social independence, and decision-making). Convergent validity was also conducted by associating the SWPER index with three outcomes: modern contraceptive use, institutional delivery, and stunting (Ewerling et al., 2017). Convergent validity is a type of construct validity used to demonstrate significant correlation between a new scale and existing validated scale; both scales should measure the same construct, thus making them theoretically identical (Netemeyer et al., 2003; Dmitrienko et al., 2007). Although associated with these three outcomes, women's empowerment is theoretically different from modern contraceptive use, institutional delivery, and stunting. Furthermore, the three outcomes do not fully describe women's empowerment and may not adequately validate the SWPER index.

Therefore, available measures of women's empowerment in sub-Saharan Africa (SSA) have several drawbacks: they lack external validity, have few domains of empowerment, and/or are not generalizable to sub-Saharan Africa. Building upon Kabeer's (2005) definition, research has identified different multidimensional and contextually relevant indicators of women's empowerment. Indeed, empirical research lists several measures of women's empowerment such as agency, autonomy, capacity for action, self-determination, and self-confidence (e.g., Cheston and Kuhn, 2001; Malhotra et al., 2002; Narayan, 2005; Hansen, 2015). These definitions stress that women's empowerment is a multifaceted concept and propose that empowerment is a process from being un-empowered to becoming empowered. Combining these views, we propose that women's empowerment is a multifaceted process of change that involves individual and collective awareness, behavior, institutions, and outcomes embedded in distinct social and cultural contexts. The goal of this study was therefore to develop a valid measure of women's empowerment both generalizable to sub-Saharan Africa and robust enough to develop region-specific indicators.

## Materials and methods

Data were extracted from the DHS in June of 2015 from countries with data available from the previous 4 years in sub-Saharan Africa. All data were from Phases 5 and 6 of the survey. The DHS characteristics and administration procedures have been previously described (ICF International, 2015). There were DHS data for 42 countries, but only 23 countries recent surveys were conducted between 2011 and 2014. DHS Data from the Republic of Congo, Guinea, Liberia, and Senegal were excluded because of missing values. Single, widowed, divorced, and separated women were excluded as most questions on women's empowerment were asked only of married women and women living with a partner. Of the remaining 167,163 partnered-women, those with missing entries on variables included in this study were excluded from the study using listwise deletion. The prevalence of women with complete data after listwise deletion was 66.6%. Finally, 19 countries were retained and classified into one of the following regions: (1) Central Africa: Cameroon, Democratic Republic of Congo, and Gabon; (2) East Africa: Comoros, Ethiopia, and Uganda; (3) Southern Africa: Mozambique, Namibia, Zambia, and Zimbabwe; and (4) West Africa: Benin, Cote d'Ivoire, Gambia, Ghana, Mali, Nigeria, Sierra Leone, and Togo. Overall, the final analysis was limited to 111,368 partnered-women with complete information on the variables used in the study (Table 1).

**Table 1:** Distribution of respondents by region and country.

**Region in Africa**	**Country/survey**	**Total women sample (15–49 years)**	**Selected women sample**
Central (*n* = 16,047)	Cameroon 2011	15,426	3,270
	Congo (Democratic Republic) 2014	10,819	9,233
	Gabon 2012	8,422	3,544
East (*n* = 11,993)	Comoros 2012	5,329	1,733
	Ethiopia 2011	16,515	6,742
	Uganda 2011	8,674	3,518
Southern (*n* = 19,683)	Mozambique 2011	13,745	5,529
	Namibia 2013	10,018	2,801
	Zambia 2014	16,411	6,538
	Zimbabwe 2011	9,171	4,815
West (*n* = 63,645)	Benin 2012	16,599	7,525
	Cote d'Ivoire 2012	10,060	4,277
	Gambia 2013	10,233	5,441
	Ghana 2014	9,396	3,822
	Mali 2013	10,424	6,397
	Niger 2012	11,160	7,158
	Nigeria 2013	38,948	20,358
	Sierra Leone 2013	16,658	4,628
	Togo 2014	9,480	4,039
All countries	Sub-Saharan Africa	247,888	**111,368**

*The number in bold represents the total number of participants included in this study*.

### Data access and ethical considerations

Access to the data sets was officially granted by the DHS program after submitting a request outlining the purpose of the analyses (https://dhsprogram.com/data/new-user-registration.cfm). The DHS contains de-identified secondary data and is considered exempt under University of Arizona's human subjects review.

### Empowerment indicators

Previous studies have suggested four important dimensions of women's empowerment in developing nations at the household level: economic, socio-cultural, education, and health (Jennings et al., 2014; Pratley, 2016). We identified several variables within each of these four dimensions and scores were assigned, with higher values reflecting greater level of empowerment. The scoring process was developed using evidence from previous literature (Kishor and Gupta, 2009; Jennings et al., 2014; Shimamoto and Gipson, 2015; Phan, 2016). Appendix A summarizes the aggregation rules used to code all variables and create the four broad dimensions and 10 overall domains.

### Indicators for economic dimension

This dimension includes labor force participation domain, created from the following indicators: respondent's occupation, type of earning from respondent's work, seasonality of respondent's occupation, and income ratio. Respondents' occupation was described by the following scores: 1—if they worked for a family member; 2—if they worked for someone else; and 3—if they were self-employed. Women's earnings were depicted using the following scores: 1—if they were paid in-kind only; 2—if they were paid cash and in-kind; 3—if they were paid in cash only. The seasonality of a woman's job was coded as follows: 1—if the woman worked occasionally or seasonally; 2—if the woman worked all year. Women's incomes were compared to their partners', and they were assigned the following scores: 1—if their partner did not bring in any income; 2—if they earned less than their partner; 3—if they earned about the same as their partner; 4—if they earned more than their partner. Women were scored 0 if they were unemployed.

### Indicators for socio-cultural dimension

This dimension includes: domains of household decision-making, attitude toward violence, life course indicator, and land or home ownership. Participation in decision-making was assessed by three items, namely: (1) person who decides respondent's healthcare; (2) person who decides large household purchases; and (3) person who decides whether respondent can visit her family or relatives. Women were assigned the following scores: 0—if the decision was made by husband/partner alone, someone else, or other; 1—if the decision was jointly made by respondent and her husband/partner; 2—if the respondent alone made the decision.

Attitudes toward violence were assessed using five variables describing whether beating was justified if the wife: goes out without telling her husband; neglects the children; argues with her husband; refuses sex with her husband; burns food. Women who answered “Yes” and “Don't know” were scored 0 while women who responded “No” were scored 1. The life course domain which was measured by two indicators—age at first birth and age at first cohabitation—were scored as follows: 0, < 15-years-old; 1, between 15- and 17-years-old; 2, between 18- and 20-years-old; 3, 21-years-old and older. Finally, two indicators—home and land ownership—described the legal status of women in possessing properties. Women were assigned the following scores: 0—if they did not own a home or land; 1—if they owned a home or land jointly; 2—if they owned home or land alone only or “both alone and jointly.”

### Indicators for education dimension

This dimension comprised three domains—literacy, highest educational level, and spousal difference in educational attainment. Women's literacy was described by the following values: 0—if they could not read at all; 1—if they were able to read part of a sentence; 2—if they were able to read an entire sentence; 3—if they did not need a reading card to assess their literacy. Women's highest educational level was measured using the following scores: 0—No education; 1—primary education, 2—secondary education; and 3—higher education. Spousal/partner difference in educational level was measured by comparing the educational attainment of respondent and her spouse/partner. Women with less educational attainment than their spouse/partner were scored 0 while those who had equal or greater educational attainment than their spouse were given 1 or 2, respectively.

### Indicators for health dimension

This dimension includes the sex negotiation and access to healthcare domains. Women's ability to negotiate sex was measured by indicators describing if they could refuse sex or ask their partner to use a condom. Women were scored 0 if they could not refuse sex or ask their partner to use a condom; otherwise, women were scored 1. Access to healthcare was classified by four indicators examining the difficulty in getting medical help, namely: (1) receiving permission before getting medical help; (2) having money for healthcare; (3) distance to health facility; (4) not wanting to go healthcare facility alone. Women were assigned a 0 score if they reported problems accessing healthcare; otherwise, respondents were scored 1.

### Data analysis

Data analysis was conducted in four main steps using STATA Version 13.1 (Stata Corporation, College Station, TX). First, the 26 retained variables in the present study were operationalized to make them eligible for factor analysis. Before running any analysis procedure, the correlation matrix was examined to justify undertaking the factor analysis. The χ^2^ for the Bartlett test of sphericity was significant at alpha = 0.01, and the Kaiser–Meyer–Olkin test showed a score of 0.80, indicating that the correlation among the variables was sufficiently strong for a factor analysis. Next, we randomly split the data (by region) into two datasets: one for EFA and the other for CFA within each region, as recommended by Worthington and Whittaker (2006), Cabrera-Nguyen (2010) and Fokkema and Greiff (2017). Therefore, we conducted exploratory and confirmatory analyses on two separate datasets in each region. Given the contextual nature of women's empowerment (Shimamoto and Gipson, 2015), all the factor analyses (EFA and CFA) were performed to identify the possible underlying factors and verify the factor structure for each region of sub-Saharan Africa.

Because our data includes categorical variables, a factor analysis was performed using a polychoric correlation matrix (Holgado-Tello et al., 2010). Afterwards, we used the “factormat” command to conduct an EFA using the matrix as input rather than raw variables. The number of factors retained was based on three criteria: (i) the Kaiser criterion (eigenvalues >1); (ii) inflection point of the screen plot; and (iii) interpretability of factors (Costello and Osborne, 2005; Suhr, 2006). Furthermore, an oblique rotation was used over orthogonal rotation because of observed correlation among factors Holgado-Tello et al. (2010).

Subsequently, we conducted a CFA on the remaining half of the randomly-split data to validate the hypothesized domains from the EFA. The CFA provided fit indices about the appropriateness of the model based on the covariance structure of the observed data such as root mean square error of approximation (RMSEA), standardized root mean square (SRMS), Bentler comparative fit index (CFI), and Tucker-Lewis index (TLI) (Schreiber et al., 2006). These modification indices explored how the model might be adjusted to improve its fit. Finally, we used the Lagrange test to derive a precise model by considering co-variance between variable in the same domain. When the co-variance was >100, we removed the items with lower EFA factor loading, resulting in the final set of variables (West Africa, *n* = 14; Central Africa, *n* = 13; East Africa, *n* = 10; and Southern Africa, *n* = 12) of included questions. The basis of such techniques is to explicitly penalize overly complex models and/or to test the model's ability to generalize by evaluating its performance on a set of data not used for EFA, which is assumed to approximate the typical unseen data that a model will encounter. The initial and final models with fit-statistics are described in Appendix B.

## Results

**Figure 1:**
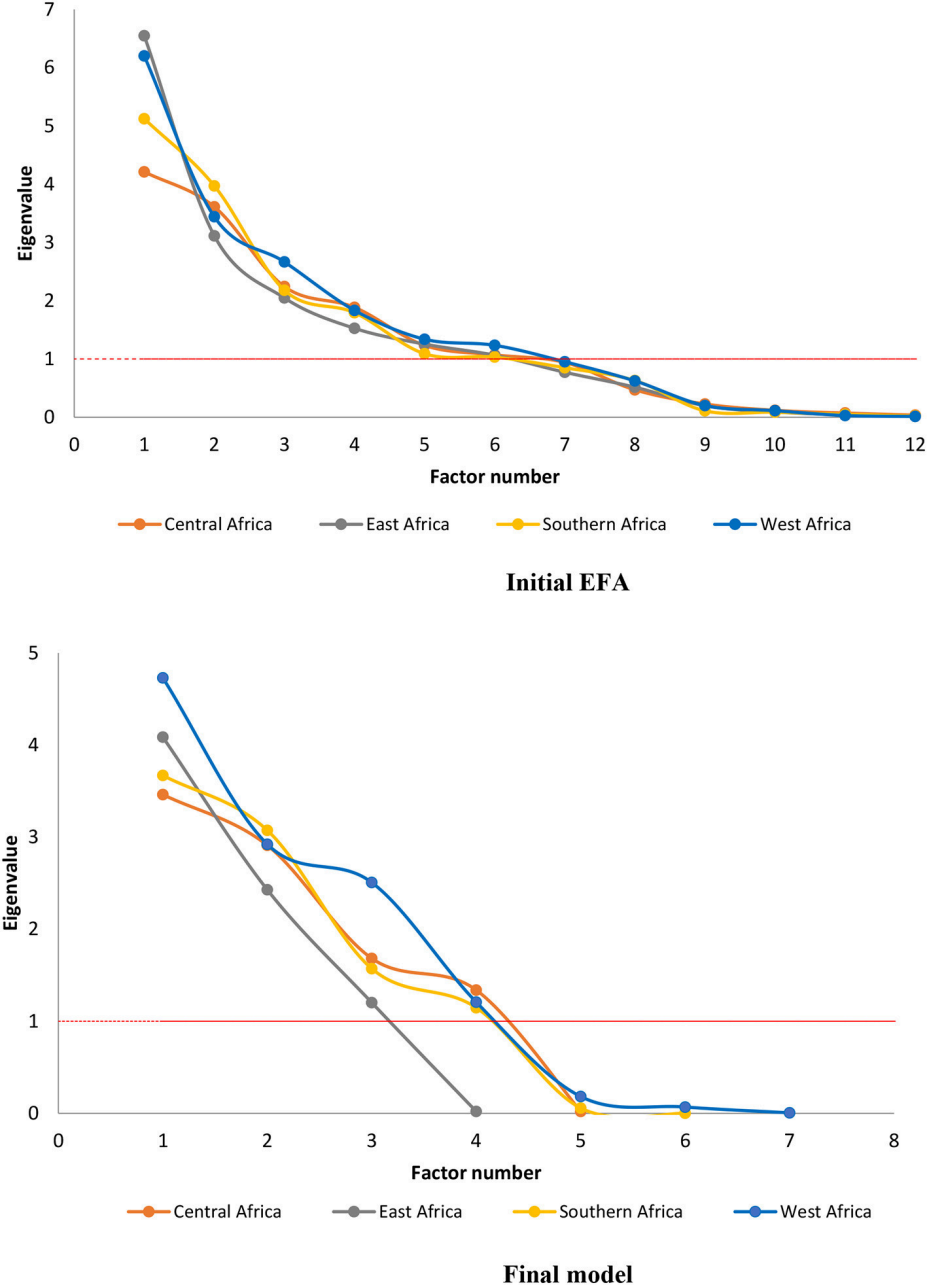
Scree plots of the initial EFA and the final model (after CFA and revision).

Scree plots showing the eigenvalues of the underlying factors derived from EFA are shown in Figure 1, with initial EFA by region shown on the initial EFA by region shown on the top and final structure (after CFA and model refinement) on the bottom. The 10 underlying factors submitted to EFA were refined down to four factors in Central Africa, Southern Africa, and West Africa and three factors in East Africa. In all regions, the first two factors accounted for most of the variation in the sample (53.4–61.2%, data not shown).

**Figure 2:**
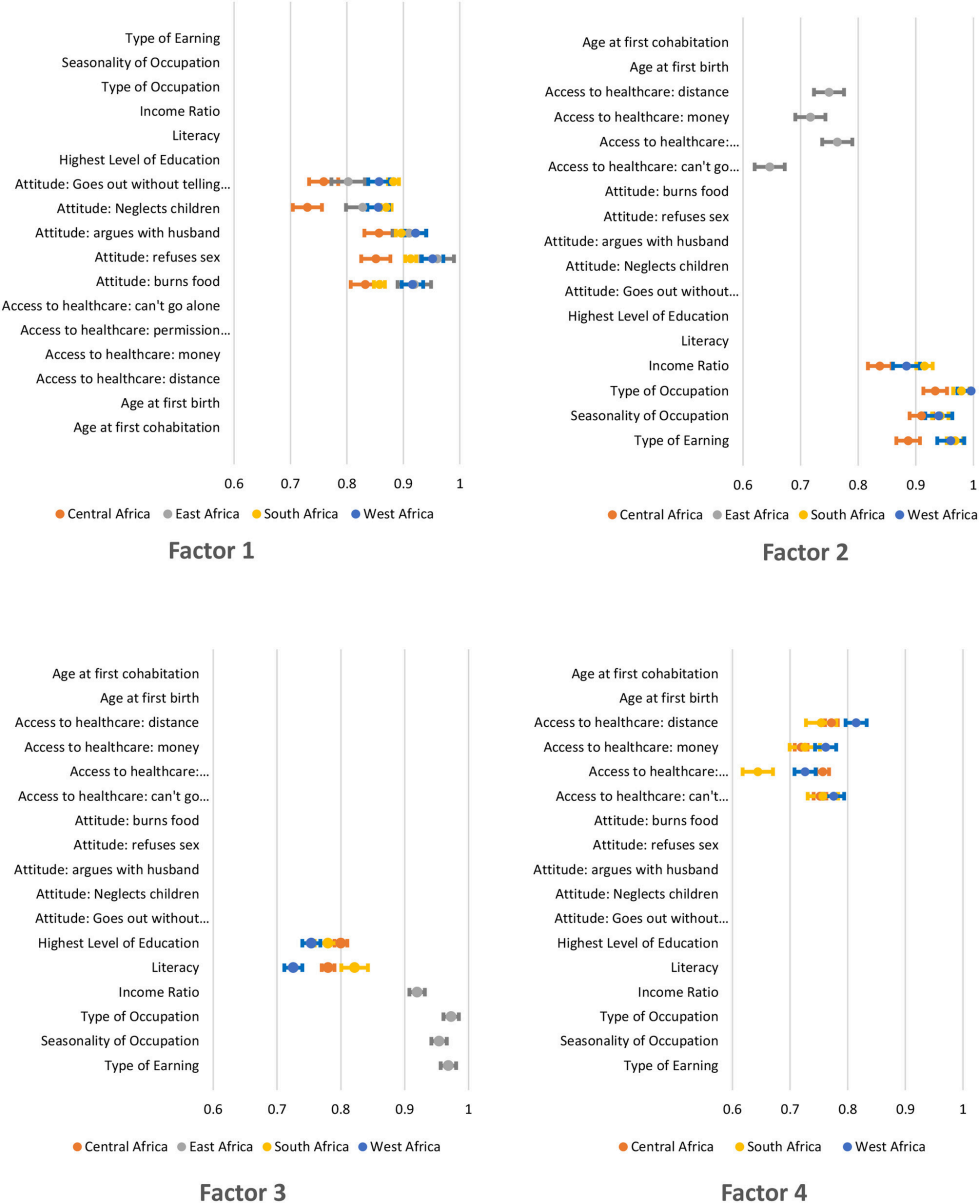
Factor loadings for independent EFA analyses.

Based on factor loadings from final EFA analyses in Figure 2, the underlying domains that contribute to women's empowerment include attitude toward violence, labor force participation, education, and access to healthcare. Specifically, the first factor loaded on attitude toward violence indicators in all four regions. In Central, Southern, and West Africa, labor force participation items lined up in the second factor, items describing education loaded on the third factor, and variables representing access to healthcare items clustered in the fourth factor. In East Africa, however, the second and third factors loaded on access to healthcare indicator and labor force participation respectively. Education did not emerge as a factor of women's empowerment in East Africa.

Within the individual factors, loadings stayed consistent between regions, but a few changed ranks within a given factor, resulting in different set of variables in the final model (Table 2). For example, education consists of two variables (literacy and highest level of education) in Central, Southern and West Africa—the regions where the domain contributed to women's empowerment. However, individual factor loadings for attitude toward violence, labor force participation, and access to healthcare varied between regions. In Central and Southern Africa, attitude toward violence consisted of four items (goes out with telling partner, neglects children, argues with husband, and burns foods) while in East Africa it comprised the first three items. In West Africa, attitude toward violence consists of four items: refuses sex, neglects children, argues with husband and burns food. Similarly, labor force participation includes type of earning, seasonality of occupation, and type of occupation for Southern Africa while it consisted of income ratio for other regions.

**Table 2:** The four domains for empowerment as determined by exploratory factor analysis (latent variables analysis), with final composition after confirmatory factor analysis and model refinement by regions.

**Domains**	**Central Africa**	**East Africa**	**Southern Africa**	**West Africa**
Attitude toward violence	Goes out with telling partner	Goes out with telling partner	Goes out with telling partner	Neglects children
	+Neglects children + Argues with husband +Burns food	+ Neglects children + Argues with husband	+ Neglects children + Argues with husband + Burns food	+ Argues with husband + Refuses sex + Burns food
Work/labor force participation	Type of earning + Seasonality + Type of occupation + Income ratio	Type of earning + Seasonality + Type of occupation + Income ratio	Type of earning + Seasonality + Income ratio	Type of earning + Seasonality + Type of occupation + Income ratio
Education	Literacy + Level of education		Literacy + Level of education	Literacy + Level of education
Access to healthcare	Cannot go alone + Needs permission + Distance	Cannot go alone + Needs Permission + Distance	Cannot go alone + Needs money + Distance	Cannot go alone + Needs permission + Needs money + Distance

## Discussion

This is the first study to use exploratory and confirmatory factor analyses to identify and validate domains of women's empowerment across the extensive sociocultural and demographic diverse regions of sub-Saharan Africa. The study emerged three valid factors of women's empowerment in East Africa, and four valid factors in Central, Southern, and West Africa.

This study builds upon existing studies (Jennings et al., 2014; Phan, 2016; Ewerling et al., 2017) by employing an extensive number of variables to describe a region-specific and validated measure of women's empowerment. In particular, this study expands on the SWEPR index by using a comprehensive list of indicators and domains of empowerment; Ewerling et al. (2017) used 15 variables in the principal component analysis in contrast with 26 variables used in our exploratory factor analysis (Ewerling et al., 2017). Although there is some overlap between the SWEPR index and this study's measure of empowerment (i.e., variables describing attitudes toward violence and education), the current study presents “access to healthcare” and “labor force participation” as validated indicators of women's empowerment. In addition this study provides region-specific indicators of women's empowerment across sub-Saharan Africa. East Africa had three indicators of women's empowerment, with “labor force participation” loading as the last indicator. In Central, Southern, and West Africa, however, there were four indicators of women's with “labor force participation” loading as the second factor. Furthermore, this study used EFA to refine the specific indicators of women's empowerment from 10 to 4 domains. Finally, this study used an appropriate validation methodology, CFA, to validate the proposed measures of women's empowerment across different regions of sub-Saharan Africa.

The domain describing women's attitudes toward violence emerged as the first and persistent factor of women's empowerment across all four regions. A substantial proportion of African women and men alike justify violence against women as evidenced in a systematic review of intimate partner violence studies conducted in three of the four African regions: Ghana, Kenya, Nigeria, Uganda, Zambia, and Zimbabwe. The review demonstrated that both sexes justified violence if woman burns food, neglects child, agues with or insults, or accuses partner of infidelity (Waltermaurer, 2012). With a high prevalence of violence justification, it is not surprising that many women experience intimate partner violence in African nations. In a recent publication, prevalence estimates of physical or sexual intimate partner violence ranged from 11.5% in Burkina Faso, 25.9% in Ivory Coast, 33.1% in Mozambique, and up to 35.2% in Zimbabwe (Peterman et al., 2015). Violence against women and girls can impede their health and socio-economic development. For instance, child brides are less likely to receive antenatal care and more likely to live in poor households (UNICEF, 2015). Women who experience intimate partner violence are at risk of poor pregnancy outcomes (Hill et al., 2016), depression, anxiety, and posttraumatic stress disorder (Lagdon et al., 2014), and sexually transmitted infections including HIV (Li et al., 2014). Therefore, the influence of violence intersects with another empowerment domain, health.

Labor force participation also emerged as a prominent indicator, suggesting that women's economic empowerment remains salient to development. These results are consistent with a similar study of empowerment in Southeast Asia (Phan, 2016). Across sub-Saharan Africa, women's economic empowerment is stalled by low educational attainment among women, cultural practices that place the burden of domestic work on women and girls, customs that inhibit women from owning lands and properties, and workplace sexual harassment (Ouida et al., 2017). These challenges put women at a disadvantage in terms of career advancement, skill acquisition, access to markets, and scaling up successful jobs. Consequently, African women are less represented in international trade ventures such as exporting cash-crops despite the fact they are over-represented in small-scale farming (UN, 2016). Thus, to promote women's economic empowerment, there must be concerted efforts promoting business enterprise. Specifically, microfinance, local savings groups, and community banks can support poor African women who may not possess collateral for loans from advanced financial institutions (van Rooyen et al., 2012). Also, the scaling of successful small and medium scale businesses should be supported by advanced financial institutions and favorable trade policies. Supporting women's economic empowerment through microfinance, international trade ventures, and effective policies result in increased “achievements”, the last arm of empowerment (UN, 2016; Ouida et al., 2017).

Access to healthcare was also a significant contributor of women's empowerment. The variables describing the access to healthcare domain—distance, money, and permission—embody the crux of empowerment, i.e., whether women have the “access” to make beneficial health choices. Financial constraints can cause all three forms of delay highlighted by Thaddeus and Maine, i.e., delay in seeking care, getting to a medical facility, and receiving care. Furthermore, farther distance, poor road conditions, or unreliable/no transportation could result in delayed or no care (Thaddeus and Maine, 1994). Improved health outcomes can be predicted by national healthcare financing. Unfortunately, public health expenditure is <4% of gross domestic product across several African countries (World Health Organization, 2013). In general, nations with limited governmental investment in health tend to have high out-of-pocket expenses, constituting an impediment to receiving healthcare. The impact of health financing on health outcomes is evident by the inverse association between total health expenditure per capita and maternal mortality ratio and under-5 mortality rate (World Health Organization, 2013). East African countries including, Ethiopia, Kenya, and Rwanda, have been noted for reducing barriers to healthcare through the provision of national health insurance scheme, increased governmental for healthcare, or utilizing community health workers in expanding health services (EIU, 2017). Nonetheless, many African countries cannot ensure accessible healthcare for their citizens, causing most Africans to still pay out-of-pocket for their medical expenses (World Health Organization, 2013). It is estimated that 11 million Africans become impoverished because of exorbitant out-of-pocket expenses (EIU, 2017). It is therefore important for African governments to foster health and healthcare through increased spending on health, training of health workers, and implementation of universal health coverage. Removing barriers to healthcare empowers women by providing resources for which women can exercise their agency.

Surprisingly, there were regional differences in four domains of women's empowerment. First, the justification of spousal violence “when women refuse sex” was pertinent to only West Africa. Despite evidence for African women condoning spousal violence pertaining to women's refusal of sex (Mugweni et al., 2015), many West African women still justify this form of violence (Uthman et al., 2009; Dako-Gyeke, 2013). The only available study on the diverse forms of spousal violence justification showed that 27.8, 29.7, and 37.6% of women in Liberia, Nigeria, and Burkina-Faso justified wife beating when a woman refuses sex with her husband (Uthman et al., 2009). Second, although education was a prominent factor of women's empowerment across other regions, it did not emerge for East Africa. This discrepancy may be explained by the fact that indicators of education in Comoros, Ethiopia, and Uganda vary vastly with 42, 49, and 73% of women aged 15–24 completing their primary education in Ethiopia, Uganda, and Comoros respectively (UNEESCO, 2017). A similar trend was also observed for average years of education; women in Ethiopia have lower years (5.1) of education than women in Ugandan (6.1 years) and Comoros (8.1 years) (UNEESCO, 2017). Therefore, with Ethiopia performing poorer than Comoros and Uganda, there may be a poor correlation between education variables and the latent construct of women's empowerment in East Africa. Third, in West Africa, the refined model excluded “age at first cohabitation” and “age at first birth,” leaving behind only indicators of education. These variables may have dropped out due to their high correlation with education which is associated with marriage and childbirth; West African girls often stop attending school once they are married and/or pregnant while those in school are less likely to be married and pregnant (UNICEF, 2015; Wodon et al., 2017).

## Limitations

There are two primary limitations to this study. First, due to the DHS's study design, this study could not assess women's cultural perception of empowerment. It is possible that women represented in these surveys have a different perception of (un)empowerment than results presented in this study. Second, because most of the indicators of women were applicable to only partnered women, this study could not explore empowerment among single, widowed, divorced, or separated women. Another limitation is our inability to include more nations in the analyses. Precisely, East Africa had only three countries, which limits the generalizability of these findings to the region.

## Conclusion and global health implications

This work builds upon the growing body of literature using the DHS datasets to define indicators for women's empowerment. This study improved upon previous indicators in several key ways. First, it is the first to utilize exploratory and confirmatory factor analyses in describing and validating the structure of women's empowerment across sub-Saharan Africa. Second, it expanded the number of domains used in previous studies (Jennings et al., 2014; Alaofè et al., 2017; Ewerling et al., 2017). Three important factors were identified commonly across the regions; attitude toward violence, economics, and access to healthcare. Third, it explored regional level differences important to the cultural and economic context of sub-Saharan Africa. These regional level analyses revealed important variation; education emerged as a factor of empowerment in Central, Southern, and West Africa. This study has overcome the shortcomings of current indices that mainly focus on developed countries, and recently on Asian countries where the perceptions of attitude toward violence and labor force participation may differ from that of sub-Saharan Africa. This validated measure is a useful tool for global health researchers in assessing the impact of women's empowerment on health outcomes. The measure also allows for comparisons across different countries and regions of sub-Saharan Africa while accounting for unique characteristics of each context.

## Author contributions

IA, HA, JG, and KE: Conceptualized the research. IA and HA: Research methodology was developed, Data analysis was conducted. IA, HA, and KE: Interpretation of Results was done. IA, HA, AA, and AM: The original manuscript was drafted. JG, JE, MH, and KE: Review and edits of manuscripts were prepared.

### Conflict of interest statement

The authors declare that the research was conducted in the absence of any commercial or financial relationships that could be construed as a potential conflict of interest.

## Footnotes

^1^UNDP, (n.d.)###UNDP (n.d.)United Nations Development Programme. *Gender Development Index*. http://hdr.undp.org/en/content/gender-development-index-gdi.v. (Accessed January 17).

^2^UNDP, (n.d.)###UNDP (n.d.)United Nations Development Programme. *Gender Inequality Index*. http://hdr.undp.org/en/content/gender-inequality-index-gii. (Accessed January 17).
