# Supplementary material for: Measuring Women's Empowerment in Sub-Saharan Africa: Exploratory and Confirmatory Factor Analyses of the Demographic and Health Surveys
Source: Front Psychol. 2018 Jun 19;9:994. doi: 10.3389/fpsyg.2018.00994 (PMC6018402; doi:10.3389/fpsyg.2018.00994)
Supplement: Supplementary file 1 [file Table_1.doc]

**Appendix A:** Domains, indicators and aggregate codes of variables used in describing women’s empowerment

| **Dimensions** | **Domains** | **Indicator label** | **Questions** | **DHS response categories** | **Recode used in analysis** |
| --- | --- | --- | --- | --- | --- |
| **Economic** | Work/Labor Force Participation | Occupation | Respondent works for family, others, self | 1 Family member; 2 Someone else; 3 Self-employed | Code 0=Not working; 1 if Q=1; 2 if Q=2; 3 if Q=3; |
|  |  | Earning | Type of earnings from respondent's work | 0 Not paid; 1 Cash only; 2 Cash and in-kind; 3 In-kind only | Code 0 if Q=0 or Not working; 1 if Q=3; 2 if Q=2; 3 if Q=1 |
|  |  | Seasonality | Respondent employed all year/seasonal | 1 All year; 2 Seasonal; 3 Occasional | Code 0 if Q=Not working; 1 if Q=2 or 3; 2 if Q=1 |
|  |  | Income Ratio | Respondent earns more than husband/partner | 1 More than him; 2 Less than him; 3 About the same; 4 Husband/partner has no income; | Code 0 if Not working; 1 if Q=4; 2 if Q=2; 3 if Q=3; 4 if Q=1 |
| **Socio-Cultural** | Household Decision-making | Women's health | Person who usually decides on respondent's healthcare | 1 Respondent alone;  2 Respondent and husband/partner;  4 Husband/partner alone;  5 Someone else;  6 Other. | Code 0 if Q= 4, 5, or 6; 1 if Q=2; 2if Q=1 |
|  |  | Large household purchases | Person who usually decides on large household purchases |  |  |
|  |  | Visiting relatives/family | Person who usually decides on visits to family or relatives |  |  |
|  | Attitudes towards violence | Goes out without telling husband | Beating justified if wife goes out without telling husband | 1. No; 2. Yes;   8 Don't know | Code 1 if Q=0; Code 0 if Q=1 or 8 |
|  |  | Neglects children | Beating justified if wife neglects the children |  |  |
|  |  | Argues with husband | Beating justified if wife argues with husband |  |  |
|  |  | Refuses sex | Beating justified if wife refuses to have sex with husband |  |  |
|  |  | Burns food | Beating justified if wife burns the food |  |  |
|  | Life course indicator | Age at first birth | Age of respondent at first birth | Age in years | Code= 0 if Q < 15; 1 if Q=15 to 17; 2 if Q=18-20; 3 if Q > 21 |
|  |  | Age at cohabitation | Age at first cohabitation | Age in years | Code= 0 if Q < 15; 1 if Q=15 to 17; 2 if Q=18-20; 3 if Q > 21 |
|  | Legal status of women | Land ownership | Owns land alone or jointly | 0 Does not own; 1 Alone only; 2 Jointly only; 3 Both alone and jointly | Code 0 if Q=0; 1 if Q=2; 2 if Q=1 or 3 |
|  |  | House ownership | Owns a house alone or jointly |  |  |
| **Education** | Literacy | Literacy | Literacy | 0 Cannot read at all; 1 Able to read only parts of sentence; 2 Able to read whole sentence | DHS Code used |
|  | Educational level | Educational level | Highest educational level | 0 No education; 1 Primary; 2 Secondary; 3 Higher | DHS Code used |
|  | Spousal difference in completeness of level of education | Spousal difference in education | Educational attainment | 0 No education; 1 incomplete primary; 2 complete primary; 3 incomplete secondary; 4 complete secondary; 5 higher  Formula: Respondent’s —Husband/Partner's education | Code:  0=respondent is less educated than partner  1=Respondent and partner have same educational attainment  2= Respondent is more educated than partner |
|  |  |  | Husband/partner's educational attainment |  |  |
| **Health** | Negotiating sex | Can ask partner to use condom | Respondent can ask partner to use a condom | 0 No; 1 Yes; 8 Don't know/not sure/depends | Code 0 if Q=0 or 8; 1 if Q=1 |
|  |  | Can say no to sex | Respondent can refuse sex |  |  |
|  | Access to Healthcare | Permission | Getting medical help for self: getting permission to go | 0 or 2 Not a big problem; 1 Big problem | Code1 if Q=0 or 2 ; Code 0 if Q=1 |
|  |  | Money | Getting medical help for self: getting money needed for treatment |  |  |
|  |  | Distance | Getting medical help for self: distance to health facility |  |  |
|  |  | Going Alone | Getting medical help for self: not wanting to go alone |  |  |

**Appendix B: Goodness of Fit Statistics**

|  | **Central Africa** | | **East Africa** | | **Southern Africa** | | **West Africa** | |
| --- | --- | --- | --- | --- | --- | --- | --- | --- |
|  | Initial | Refined | Initial | Refined | Initial | Refined | Initial | Refined |
| CFI | 0.963 | 0.980 | 0.978 | 0.993 | 0.976 | 0.988 | 0.932 | 0.986 |
| TLI | 0.954 | 0.974 | 0.972 | 0.991 | 0.970 | 0.984 | 0.918 | 0.982 |
| RMSEA | 0.056 | 0.047 | 0.053 | 0.037 | 0.052 | 0.040 | 0.083 | 0.043 |
| p(RMSEA) | <= 0.05 | <= 0.05 | <= 0.05 | <= 0.05 | <= 0.05 | <= 0.05 | <= 0.05 | <= 0.05 |
| SRMR | 0.0320 | 0.0240 | 0.031 | 0.023 | 0.027 | 0.022 | 0.055 | 0.024 |
| AIC | 178204.462 | 161992.977 | 103544.345 | 86724.038 | 166580.192 | 152068.138 | 766998.400 | 573188.300 |
| BIC | 178560.566 | 162307.187 | 103825.175 | 86944.690 | 166947.587 | 152370.698 | 767475.400 | 573590.000 |
| CD | 1.000 | 1.000 | 0.999 | 0.998 | 1.000 | 1.000 | 1.000 | 1.000 |

Desirable indices from **Scheiber el al., 2006**: Root mean square error of approximation, RMSEA < 0.06; Comparative Fit Index, CFI > 0.96; Tucker-Lewis Index, TLI > 0.95; Standardized Root Mean Residual, SRMR < 0.08; Coefficient of Determination (CD)
